# Effect of Material Heterogeneity on Environmentally Assisted Cracking Growth Rate of Alloy 600 for Safe-End Welded Joints

**DOI:** 10.3390/ma14206186

**Published:** 2021-10-18

**Authors:** Kuan Zhao, Shuai Wang, He Xue, Zheng Wang

**Affiliations:** School of Mechanical Engineering, Xi’an University of Science and Technology, Xi’an 710054, China; 17101016005@stu.xust.edu.cn (S.W.); xuehe@xust.edu.cn (H.X.); 19205201083@stu.xust.edu.cn (Z.W.)

**Keywords:** environmentally assisted cracking, alloy 600, crack growth rate, heterogeneous, randomness, film slip-dissolution/oxidation model

## Abstract

Environmentally assisted cracking (EAC) is essential in predicting light water reactors’ structural integrity and service life. Alloy 600 (equivalent to Inconel 600) has excellent corrosion resistance and is often used as a welding material in welded joints, but material properties of the alloy are heterogeneous in the welded zone due to the complex welding process. To investigate the EAC crack growth behavior of Alloy 600 for safe-end welded joints, the method taken in this paper concerns the probability prediction of the EAC crack growth rate. It considers the material heterogeneity, combining the film slip-dissolution/oxidation model, and the elastic-plastic finite element method. The strain rate at the crack tip is a unique factor to describe the mechanical state. Still, it is challenging to accurately predict it because of the complicated and heterogeneous material microstructure. In this study, the effects of material heterogeneity on the EAC crack growth behavior are statistically analyzed. The results show that the material heterogeneity of Alloy 600 can not be ignored because it affects the prediction accuracy of the crack growth rate. The randomness of yield strength has the most influence on the EAC growth rate, while Poisson’s ratio has the smallest.

## 1. Introduction

Due to their outstanding corrosion resistance and excellent mechanical properties in high-temperature and wet corrosive environments, nickel-based alloys are widely used as welding consumables in the safe-end welded joints [1,2]. However, the susceptibility of nickel-based alloys to Environmentally assisted cracking (EAC) in operational service has been revealed in recent years. Different types of defects, such as circumferential and axial defects, were observed, and serious leakage events were reported [3]. Because of the high cost of replacing or repairing nuclear power plants components, many countries need to look into extending their lives. However, the long-term life extension is often accompanied by potential safety hazards due to the EAC of safe-end welded joints [4]. Dozens of mechanisms and predictive models have been proposed for EAC crack propagation [5,6,7], and the slip-dissolution/oxidation model has been widely accepted as a reasonable EAC description of the critical materials in nuclear plants [8]. In the model, the strain rate at the crack tip is usually used as a unique factor to describe the mechanical condition, but it is challenging to obtain it. Xue et al. [9,10] proposed an approach for the quantitative prediction of EAC crack growth rate. In this method, the selection of characteristic distance *r*_0_ is essential. If *r*_0_ is unsuitable, the predicting results may deviate from the actual data [11]. However, it is difficult to accurately predict it because the material microstructure is complicated and heterogeneous [12].

It is well known that nickel-based alloys in welded joints are heterogeneous because of the complex welding process [13], and they continue to suffer from high temperature, high pressure, nuclear irradiation, and corrosive environment [14]. The stress and strain distributions of EAC crack tips have been investigated in many papers since they shed information on the fracture behavior of solids. Previous articles have reported that the welded joint’s strength mismatch and sampling position would observably affect the stress and strain distributions [15,16]. However, the material heterogeneity is mainly reflected in the difference in the yield strength of the welded metal and the base metal on both sides of the fusion line. The deformation imparted during welded processing is not homogenously distributed, and the inhomogeneity can lead to differences in strength, grain size, dislocation density, and corrosion resistance from the surface. Some scholars have measured the microhardness of nickel-based alloys [17,18] and found that the mechanical properties of the same material at different positions are also different. However, little information exists about how such differences influence the mechanical state of the EAC crack tip.

Since the various factors relating to inhomogeneity are random, the mechanical properties such as elastic modulus, Poisson’s ratio, and strength change with the change in the space position inside the material. To understand the effect of such heterogeneity on the EAC growth rate of Alloy 600, an approach to predict the probability of EAC growth rate is proposed and discussed in the paper. The results provide a better understanding of the EAC behavior of Alloy 600 used in safe-end welded joints.

## 2. Theoretical Model for Environmentally Assisted Cracking (EAC) Growth Rate Prediction

According to the film slip-dissolution/oxidation model, the EAC growth of nickel-based alloys could be modeled by rupture of the oxide film and the anodic dissolution process in a high-temperature oxygenated aqueous system. Ford and Andresen [19] have conducted a large number of experimental research and concluded that most EAC of alloys could be explained by the model. The specific expression of the model can be written as:

(1) $$\frac{da}{dt}=\frac{M}{Z\times \rho \times F}\times \frac{i_{0}}{1-m}\times {\left(\frac{t_{0}}{\varepsilon_{f}}\right)}^{m}\times {\left({\dot{\varepsilon }}_{\mathrm{ct}}\right)}^{m}$$

where d*a*/d*t* is the EAC crack growth rate, *M* is the atomic weight of the alloys, *Z* is the change in charge caused by the oxidation process, *ρ* is the density of the alloys, *F* is Faraday’s constant, *i*_0_ is the oxidation current density of the exposed surface, *t*_0_ is the time before the onset of the current decay, *ε*_f_ is the degradation strain of the protective film, ${\dot{\varepsilon }}_{\mathrm{ct}}$ is the strain rate at the crack tip, and *m* is the exponent of the current decay curve, which is related to corrosion potential, solution conductivity, and chromium consumption.

The model is Ford’s well-known model, widely used in studies of the EAC behavior in light-water reactors [19]. However, it is difficult to obtain ${\dot{\varepsilon }}_{\mathrm{ct}}$ at the tip exactly in practical application, so it is proposed that ${\dot{\varepsilon }}_{\mathrm{ct}}$ can be substituted by the tensile plastic strain rate ${\dot{\varepsilon }}_{p}$ at a characteristic distance *r*_0_ in front of the crack tip [20], and ${\dot{\varepsilon }}_{\mathrm{ct}}$ can be written as:

(2) $$\frac{d\varepsilon_{\mathrm{ct}}}{dt}=\frac{d\varepsilon_{p}}{dt}=\frac{d\varepsilon_{p}}{da}\times \frac{da}{dt}$$

**Figure 1:**
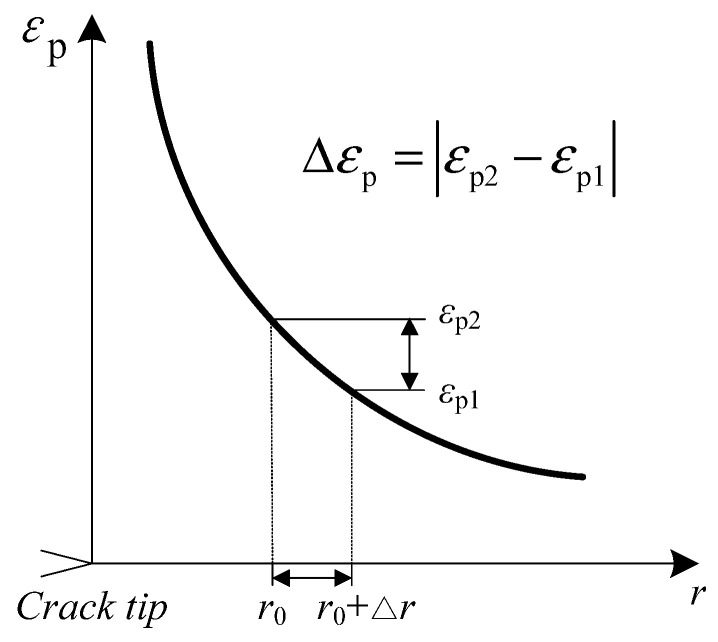
Acquisition of the plastic strain rate by numerical simulation.

According to the strain gradient theory [21], when the stress intensity factor is constant, the change of tensile plastic strain at the characteristic distance *r*_0_ in front of a crack tip can be expressed as Equation (3), and d*ε*_p_/d*a* can be calculated using numerical simulations, as shown in Figure 1.

(3) $$\frac{d\varepsilon_{p}}{da}\approx \frac{∆\varepsilon_{p}}{∆r}=\frac{\varepsilon_{p2}|{}_{r=r_{0}+∆r}-\varepsilon_{p1}|{}_{r=r_{0}}}{∆r}$$

Substituting Equations (2) and (3) into Equation (1), the EAC growth rate can be expressed as

(4) $$\frac{da}{dt}=\kappa_{a}^{\prime }\times {\left(\frac{∆\varepsilon_{p}}{∆r}\right)}^{m/\left(1-m\right)}$$

where

(5) $$\kappa_{a}^{\prime }={\left(\frac{M}{Z\times \rho \times F}\times \frac{i_{0}}{1-m}\times {\left(\frac{t_{0}}{\varepsilon_{f}}\right)}^{m}\right)}^{1/\left(1-m\right)}$$

In this model, *r*_0_ is an essential parameter. Theoretically, *r*_0_ should be obtained according to the EAC growth mechanism, but it is currently an unclear variable. Fortunately, much research on key materials in light-water reactors has been done over the past four decades using compact tension C.T. specimens. *r*_0_ could be determined quantitatively by incorporating the slow strain rate tensile test data and numerical calculations.

## 3. Calculation Model for EAC Growth Rate Prediction

### 3.1. Material and Simulation Test Conditions

Nickel-based Alloy 600 is one of the most widely used welding consumables in safe-end welded joints due to its excellent properties. Due to the particularity of the processing technique and service environment, defects such as cracks, composition gradient, impermeability, and inclusions will inevitably exist in the welded metal [22].

**Figure 2:**
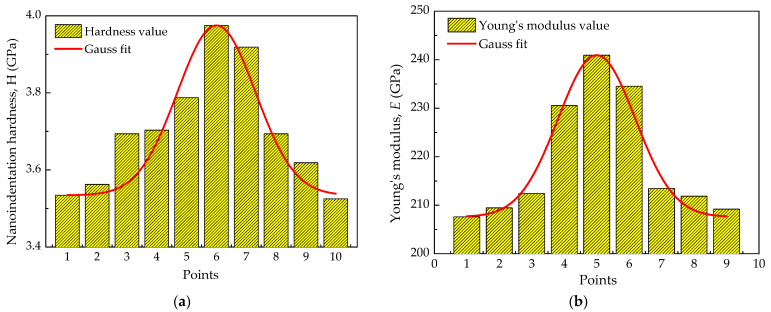
Micromechanical properties of the weld center area of the weld joint. (**a**) Nanoindentation hardness; (**b**) Young’s modulus.

Some welding experiment using nickel-based alloy (Tiantai Electrode Company, Kunshan, Jiangsu, China) as the filler material was carried out [23], and nano hardness and elastic modulus data of the welding center area were measured using the nanoindentation method. The results are shown in Figure 2. It was found that the random sample points of nano hardness and elastic modulus approximately obey the normal distribution. Based on the relation presented by Tabor [24] in 1951, which connects the yield stress of classical materials to hardness by a simple factor of proportionality, material parameters, such as yield strength, Young’s Modulus, and Poisson’s ratio of Alloy 600, are considered to obey normal distribution with an expected mean value *μ* and a coefficient of variation (*C*.*V*.) [25]. The *C*.*V*. is defined by *σ*/*μ*, in which σ is the standard deviation. These data are randomly designated, one by one, to different locations on the model as its material properties. The mean mechanical properties of the material in high-temperature water at 340 °C are shown in Table 1 [26]. The primary purpose of the study is to calculate the effect of material heterogeneity on EAC crack growth behavior. The oxidation rate constant $\kappa_{a}^{\prime }$ was 7.478 × 10^−7^, and the exponent *m* of the current decay curve was 0.5 [27].

### 3.2. Specimen and Finite Element Model

**Figure 3:**
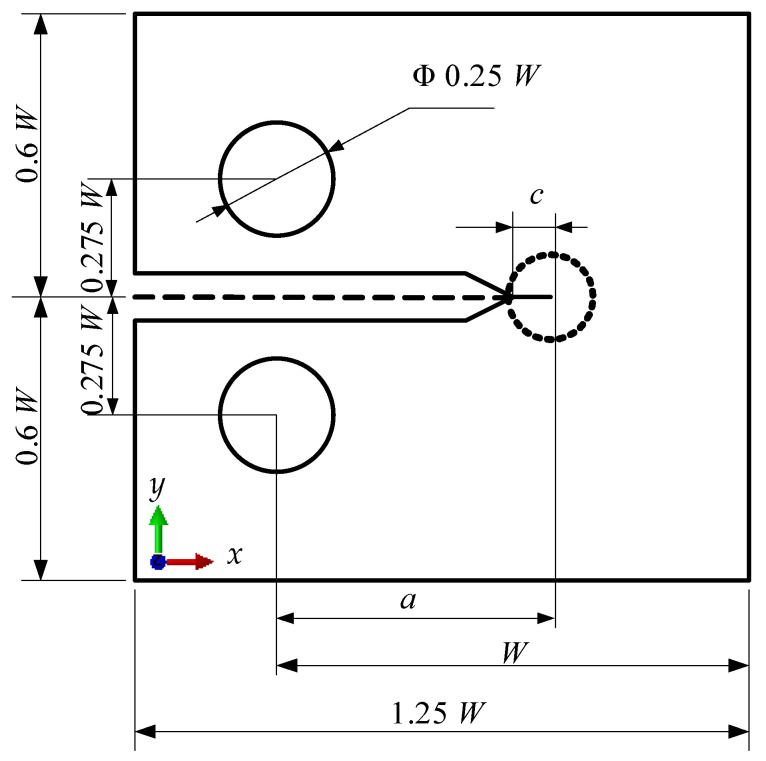
Geometric size of the compact tension specimen (where, *W* = 50 mm, *a* = 0.5 W, *c* = 1.5 mm).

The compact tension (C.T.) specimen is widely adopted in the EAC tests in high-temperature water environments. Thus, a C.T. specimen is adopted as a geometric model in this research, and the numerically simulated process is guided by the American Society for Testing and Materials (ASTM) standards [28]. The geometric shape and size of the C.T. specimen are shown in Figure 3. In the coordinate system, the *x*-coordinate direction is parallel to the crack growth, and the *y*-coordinate is perpendicular to the crack growth direction.

**Figure 4:**
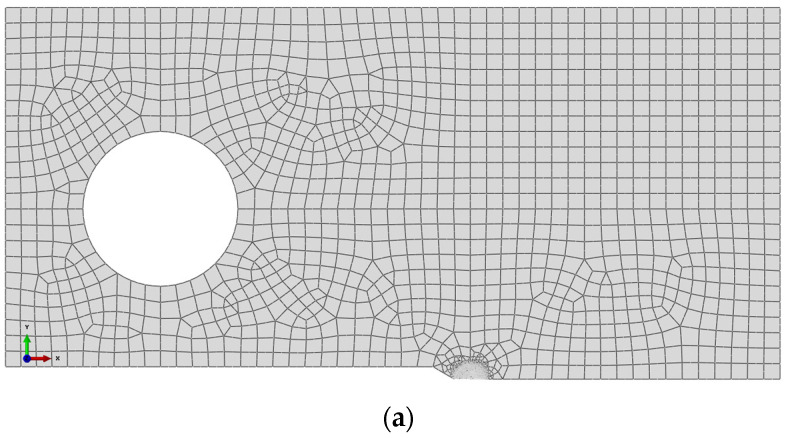

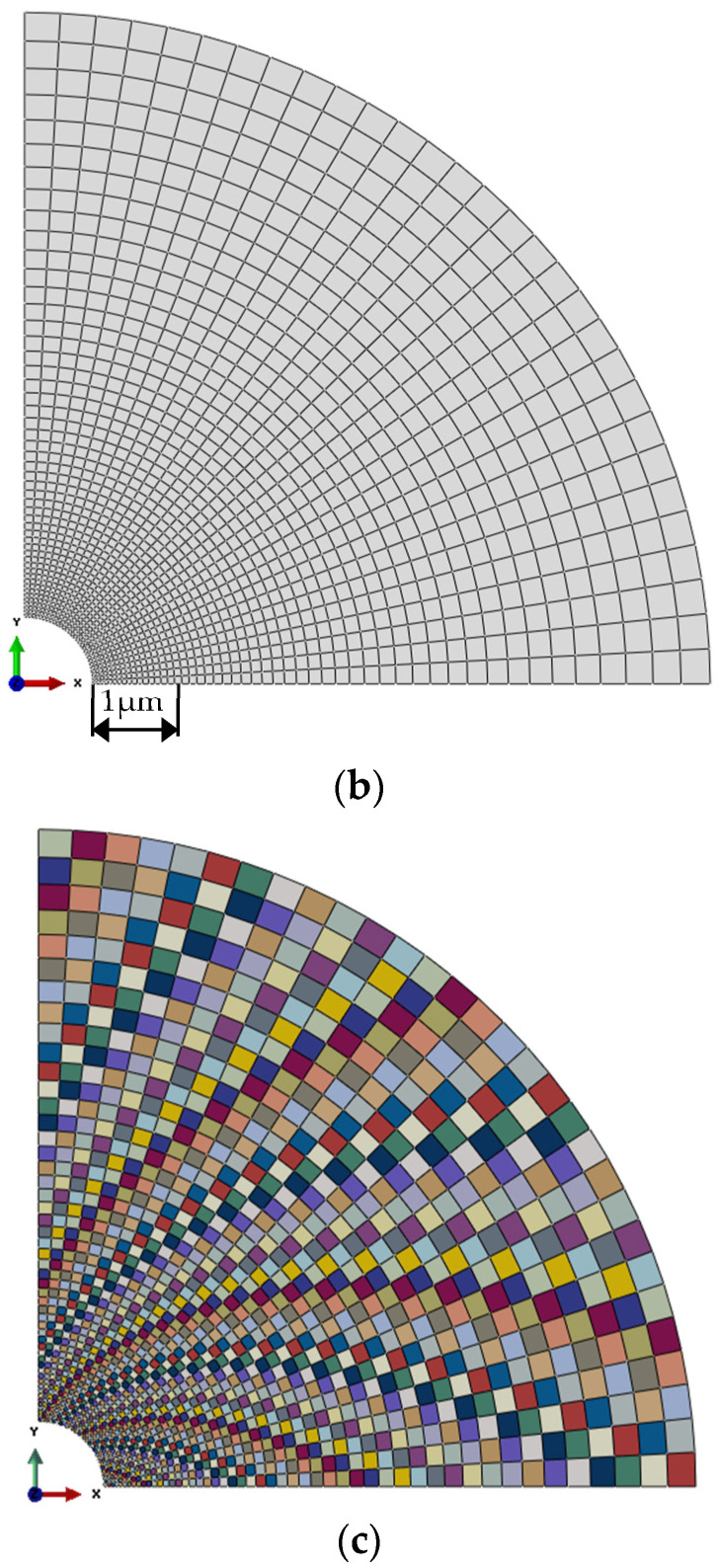
Finite element mesh of 1T-C.T. specimen: (**a**) mesh of half specimen, (**b**) detail around the crack tip of homogeneous material, and (**c**) detail around the crack tip of heterogeneous material.

The loading process is simulated by a commercial finite element code ABAQUS (ABAQUS V6.14, 2015, FRANCE/DASSAULT Company, Vélizy-Villacoublay, France), which is expected to obtain the mechanical state in the entire EAC experimental process. To investigate the local stress and strain distribution at the crack tip, a sub-model technique was adopted in the calculation. Because the crack front that runs along the thickness of the specimen is mainly dominated by the plane strain condition in the welded joints, the specimen is simplified as a plane strain model. The finite element mesh of the C.T. specimen is shown in Figure 4, where 16,086 eight-node biquadrate plane strain quadrilateral (CPE8) elements are adopted in the whole model, and 27,997 CPE8 elements are adopted in the sub-model. Considering the material heterogeneity of Alloy 600, the random distribution is realized by assigning values to different material properties of all elements, as shown in Figure 4c, in which different gray levels correspond to different material properties.

### 3.3. Load Condition

An accurate description of the stress intensity factor *K*_Ι_ at the crack tip is particularly important in the study of the crack growth rate of Alloy 600. Since the hoop stress *σ*_θ_ of the pressure pipeline is usually much greater than the axial and radial stress, it is necessary to establish the relationship between *K*_Ι_ and *σ*_θ_. According to the stress theory and the calculation formula of fracture mechanics, *σ*_θ_ and *K* can be calculated as [26]:

(6) $$\sigma_{\theta }=-\frac{PD}{2b}$$

(7) $$K_{Ι}=A\sigma_{\theta }\sqrt{\pi \alpha }$$

where *A* is the correction factor which is 1.1215 for single-edged crack. *α* is the crack length, which is assumed to be 1.5 mm because it is the shortest value in the crack test in E399. According to the designed parameters of the AP1000 reactor pressure pipeline, which has a diameter *D* of 4400 mm, a wall thickness *b* of 225 mm, and internal pressure *P* of 15 MPa, *σ*_θ_ is 147 MPa, the stress intensity factor *K*_Ι_ calculated by Equation (7) is about 11 MPa·m^1/2^. The tensile load *P* applied to the C.T. specimen can be calculated as [28]:

(8) $$K_{Ι}=\frac{P}{B\sqrt{W}}f\left(\frac{a}{W}\right)$$

where

(9) $$f\left(\frac{a}{W}\right)=\frac{\left(2+\frac{a}{W}\right)\left[0.866+4.64\frac{a}{W}-13.32{\left(\frac{a}{W}\right)}^{2}+14.72{\left(\frac{a}{W}\right)}^{3}-5.6{\left(\frac{a}{W}\right)}^{4}\right]}{{\left(1-\frac{a}{W}\right)}^{3/2}}$$

**Figure 5:**
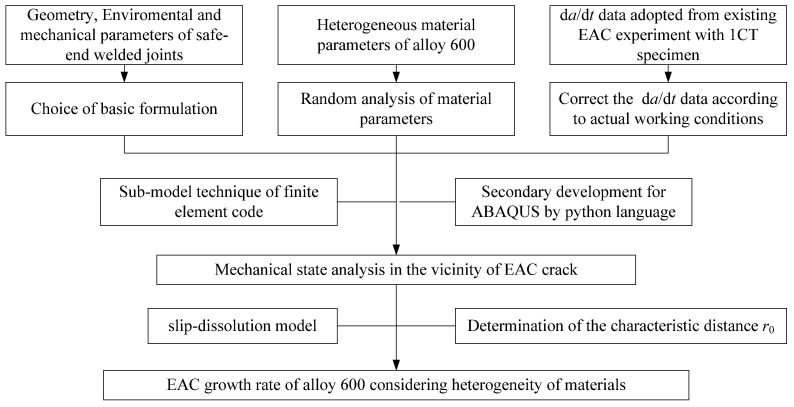
The framework of the prediction process.

The framework for the prediction process is shown in Figure 5. Firstly, the stress theory and the calculation formula of fracture mechanics are chosen and adopted, combining the geometry, environmental, and mechanical parameters of safe-end welded joints. In addition, statistical characterization is investigated by heterogeneous material parameters of Alloy 600, and d*a*/d*t* data is adopted and corrected by existing EAC experiments using the C.T. specimens. Secondly, the combination of sub-model technique of finite element code and secondary development of ABAQUS by Python language, the mechanical state in the vicinity of EAC crack is analyzed, and the characteristic distance in front of the crack tip *r*_0_ is determined. Finally, the EAC growth rate along the crack front is predicted by combining Equation (4) and characteristic distance *r*_0_, which is determined by combining experimental EAC data under the same load and test environmental conditions with a finite element analysis considering material heterogeneity.

## 4. Results and Discussion

### 4.1. Effect of Material Properties on the Normal Plastic Strain

The normal plastic strain $\varepsilon_{p}^{22}$ in front of the crack tip is often adopted as the primary mechanical parameter influencing the EAC crack growth rate prediction. To examine the mechanical state under homogeneous and heterogeneous conditions near the crack tip, the crack tip is designed with a small crack size blunt notch with a radius of 1 μm, and the *C.V.* of random variables are set to 0.2.

**Figure 6:**
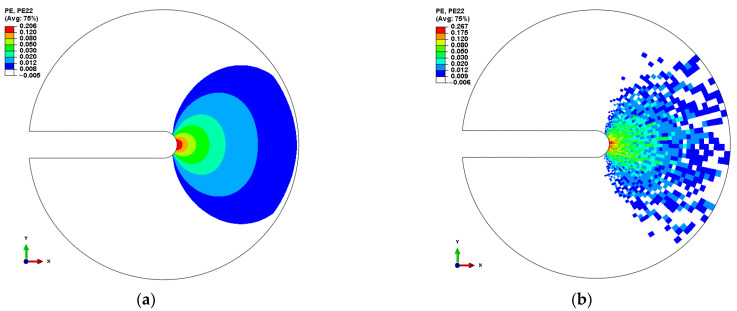
Normal plastic strain (PE22) ahead of the crack tip: (**a**) homogeneous material and (**b**) heterogeneous material.

The $\varepsilon_{p}^{22}$ distribution ahead of the crack tip under homogeneous materials and heterogeneous materials is shown in Figure 6, which shows that the maximum $\varepsilon_{p}^{22}$ is nearby the crack tip, and the $\varepsilon_{p}^{22}$ in front of the crack tip rapidly decreases with an increasing distance from the crack tip. In addition, the $\varepsilon_{p}^{22}$ variation trend of heterogeneous conditions is similar to that of homogeneous materials. Comparing Figure 6a with Figure 6b, it is evident that the heterogeneity of the materials results in the dispersion of $\varepsilon_{p}^{22}$ at the crack tip because the mechanical properties of each element are randomly distributed. More attention should be given to the heterogeneity of the materials in order to improve the prediction of the EAC growth rate.

**Figure 7:**
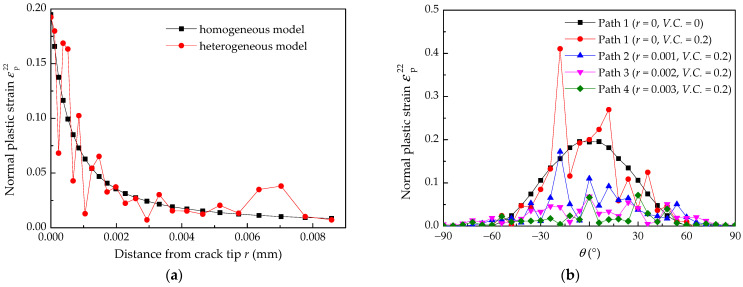
Normal plastic strain under homogeneous and heterogeneous conditions: (**a**) in front of the crack tip; (**b**) around the crack tip.

The extent of crack jumping distance is on the order of 1–10 μm [26], so the distance from the crack tip *r* was chosen to be 1–10 μm. The distribution of the normal plastic strain $\varepsilon_{p}^{22}$ in front of and around the crack tip is shown in Figure 7a. The normal plastic strain in front of the crack tip rapidly decreases with an increasing distance from the crack tip, and the $\varepsilon_{p}^{22}$ curves for the heterogeneous material fluctuate up and down along the curves of the homogeneous material, which indicates that the dispersion of material properties has a significant effect on the mechanical properties of the crack tip. In other words, the heterogeneity of the materials is not ignored because it may affect the prediction accuracy of the crack growth rate.

When the distance *r* is 0, 1, 2, or 3 μm from the crack tip, the normal plastic strain around the crack tip from −90° to 90° is shown in Figure 7b. The $\varepsilon_{p}^{22}$ variation trend of random materials is similar to homogeneous materials. The maximum $\varepsilon_{p}^{22}$ under homogeneous condition occurs along the 0° direction. Nevertheless, the maximum $\varepsilon_{p}^{22}$ under heterogeneous conditions appears around the 0° direction. It is suggested that *r*_0_ can be determined by combining experimental EAC data with a finite element analysis considering material heterogeneity.

### 4.2. Effect of a Single Random Material Parameter on the Crack Growth Rate

To compare the effects of different random parameters of Alloy 600 on the growth rate, the yield strength, Young’s Modulus, and Poisson’s ratio of Alloy 600 are separately set to a single random parameter. The coefficients of variation are simplified as *C*.*V*.*_E_*, *C*.*V*.*ν*, *C*.*V*.*_σ_*, and others are considered as deterministic parameters.

After simulating the normal plastic strain, the variation of the normal plastic strain with respect to the crack growth d*ε*/d*r* can be obtained by the calculated method represented in Figure 1. Then by substituting d*ε*/d*a* in Equation (4), the effect of randomness on the crack growth rate of Alloy 600 in the high temperature oxygenated aqueous environment can be analyzed. 

**Figure 8:**
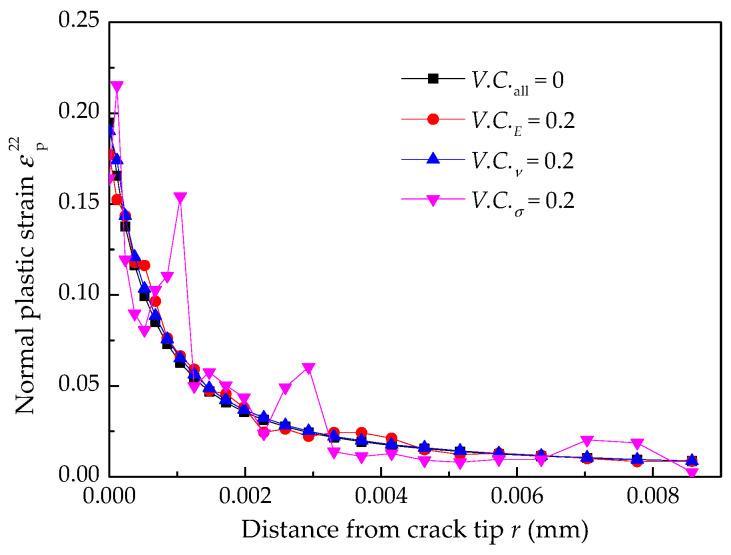
Normal plastic strain under a single random material factor.

**Figure 9:**
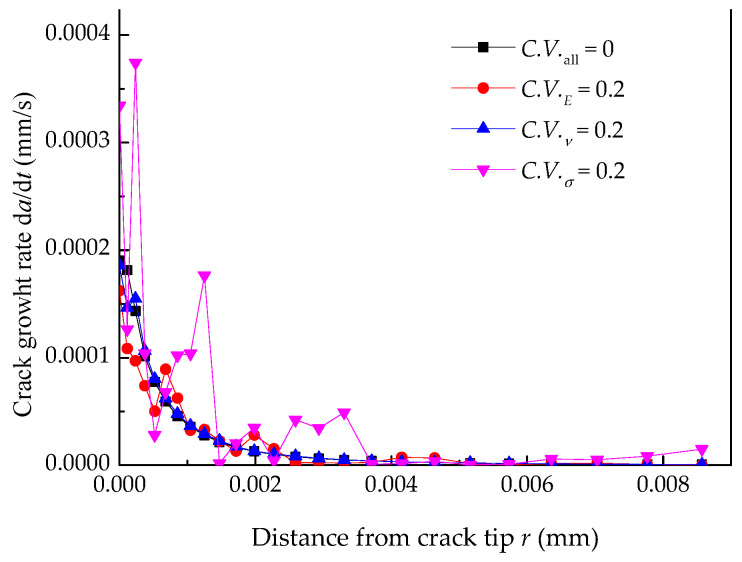
Crack growth rate under a single random material factor.

The normal plastic strain and crack growth rate under a single random material factor are shown in Figure 8 and Figure 9, indicating that the normal plastic and crack growth rate depend on the characteristic distance *r*_0_. If the appropriate value of *r*_0_ can be determined, the EAC growth rate under different material and mechanical environments can be obtained. The randomness of yield strength of Alloy 600 has the most considerable influence on the normal plastic strain and the EAC growth rate, but that of the Poisson’s ratio has the smallest.

### 4.3. Effect of Yield Strength Distribution on the Crack Growth Rate

**Figure 10:**
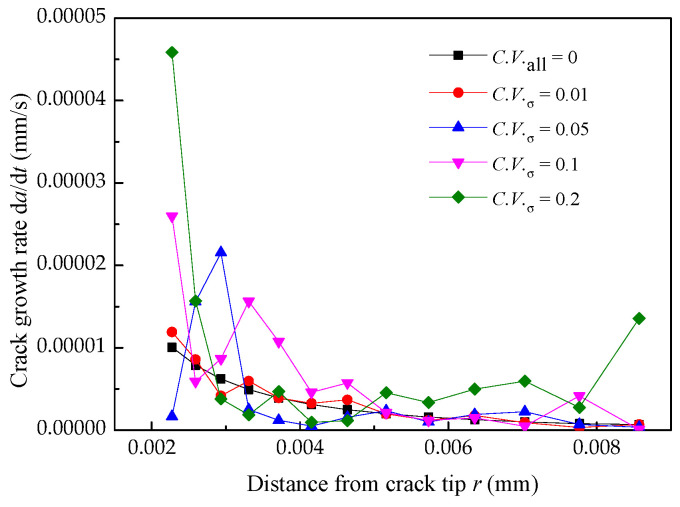
Crack growth rate under different Yield strength distribution.

Since the randomness of Yield strength has a significant effect on the crack growth rate, to investigate the influence of Yield strength distribution of Alloy 600 on the growth rate, Young’s Modulus and Poisson’s ratio are fixed as the mean values. Yield strength is assumed to obey a normal distribution with a coefficient of variation varying from 0.01 to 0.05 to 0.1 to 0.2 [29]. The effect of different yield strength distributions on the crack growth rate is shown in Figure 10, which indicates that as *C.V._σ_* increases, the dispersion of the crack growth rate increases. In other words, the more uniform the material is, the smaller fluctuation of crack growth rate will be observed.

### 4.4. Comparison of EAC Growth Rate

**Figure 11:**
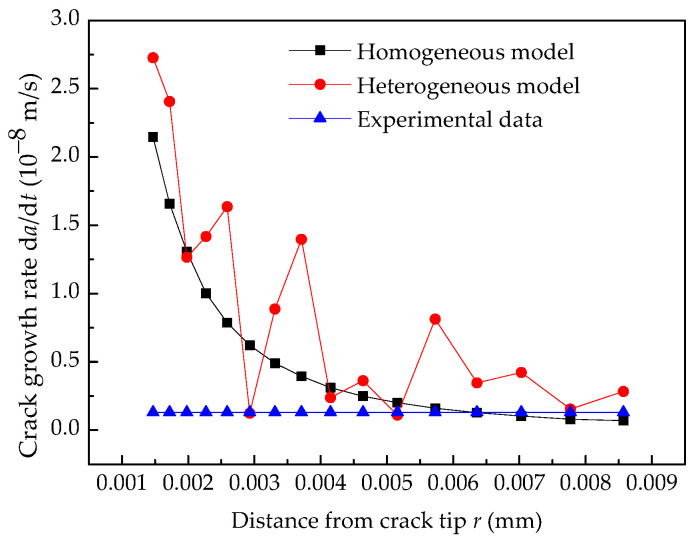
Comparison of EAC growth rate with experimental data.

The comparison of EAC growth rate among the homogeneous model, heterogeneous model, and experimental data are shown in Figure 11. In the heterogeneous model, the *C.V.* of random variables was set to 0.2. The results show that the growth rate predicted by the homogeneous model is monotonically decreasing and varies from 2.1 × 10^−8^ to 6.9 × 10^−10^ m s^−1^ when *r*_0_ changes from 1.5 to 9 μm. The growth rate predicted by the heterogeneous model declines with any fluctuations and varies from 3.2 × 10^−8^ to 2.8 × 10^−9^ m s^−1^ when *r*_0_ changes from 1.5 to 9 μm. Thus, the material heterogeneity leads to divergence from the predicted results, and the results predicted by these two models depend on the characteristic distance *r*_0_. Based on the experimental result by Rebak et al. [30], under the same load, when *r*_0_ equals 6.36 μm, the predicted results by the homogeneous model are in good agreement with the experimental results. However, the result predicted by the heterogeneous model is approximately three times the experimental result. Thus, the material heterogeneity of Alloy 600 can not be ignored because it affects the prediction accuracy of the crack growth rate. It is easy to see that 2.91 μm is a more reliable choice for *r*_0_ in the heterogeneous model.

## 5. Conclusions

By incorporating the film slip-dissolution/oxidation model and the elastic-plastic finite element method, a method is derived for predicting the EAC growth rate of Alloy 600 with heterogeneous materials for safe-end welded joints. Random distribution is realized by assigning values to different material properties of all the elements. The effects of the random parameters on the mechanical state and crack growth rate are statistically analyzed, and the method of determining the characteristic distance *r*_0_ has been further improved. The following conclusions are obtained:

1. An approach that will enable the prediction of the EAC growth rate of Alloy 600 for safe-end welded joints is proposed and discussed, considering the material heterogeneity. The approach can be adopted to develop an understanding of the EAC growth rate of Alloy 600 for safe-end welded joints.
2. The heterogeneity of the materials is not ignored because it may affect the prediction accuracy of the crack growth rate. The randomness of the yield strength has the most considerable influence on the EAC growth rate, but that of Poisson’s ratio has the smallest.
3. A characteristic distance from the crack tip *r*_0_ is a critical parameter in the approach proposed here. It is suggested that *r*_0_ can be determined by combining experimental EAC data under the same load and test environmental conditions with a finite element analysis considering material heterogeneity.

## Figures and Tables

**Table 1:** Mechanical properties of Alloy 600 in Pressurized Water Reactor environment (340 °C).

Material Parameter	Value
Young’s Modulus, *E* (MPa)	189,500
Poisson’s ratio, *ν*	0.286
Yield strength, *σ*_0_ (MPa)	436
Yield offset, *α*	3.075
Hardening exponent, *n*	6.495

## Data Availability

The data presented in this study are available on request from the corresponding author.

## Nomenclature

EAC	Environmentally assisted cracking
*C.V.*	coefficient of variation
*K*_Ι_	stress intensity factor
*a*	Actual crack size
*c*	Physical crack size including plastic zone
*M*	atomic weight of the metal
*Z*	change in charge due to the oxidation process
*ρ*	density of the metal
*F*	Faraday’s constant
*i*_0_	oxidation current density of the bare surface
*m*	exponent of the current decay curve
*t*_0_	time before the onset of the current decay
*ε*_f_	degradation strain of the protective film
${\dot{\varepsilon }}_{\mathrm{ct}}$	strain rate at the crack tip
*ε*_p_	tensile plastic strain
*r*_0_	characteristic distance
$\kappa_{a}^{\prime }$	The oxidation rate constant
*E*	Young’s Modulus
*ν*	Poisson’s ratio
*σ*_0_	Yield strength
*α*	Yield offset
*n*	Hardening exponent
*W*	Specimen width
*σ*_θ_	hoop stress of the pressure pipeline
*P*	internal pressure of pressure pipeline
*D*	diameter of pressure pipeline
*b*	wall thickness of the AP1000 reactor pressure pipeline
$\varepsilon_{p}^{22}$	Normal plastic strain
${\dot{\varepsilon }}_{p}$	tensile plastic strain rate
